# Left Ventricular Noncompaction in a Child with Turner Syndrome

**DOI:** 10.1155/2019/6824321

**Published:** 2019-11-11

**Authors:** Snigdha Bhatia, Amna Qasim, Murad Almasri, Luba Frank, Ashraf M. Aly

**Affiliations:** ^1^Department of Pediatrics, University of Texas Medical Branch, Galveston, TX, USA; ^2^Department of Radiology, University of Texas Medical Branch, Galveston, TX, USA; ^3^Division of Pediatric Cardiology, University of Texas Medical Branch, Galveston, TX, USA

## Abstract

Congenital heart disease (CHD) may cause a significant comorbidity in patients with Turner syndrome. The commonly reported CHD in these patients includes bicuspid aortic valve and coarctation of the aorta. Left ventricular noncompaction (LVNC) is a rare form of cardiomyopathy that has been reported in literature only three times in adult patients with Turner syndrome. We report the first case of a 6-year-old asymptomatic female with Turner syndrome who was referred for cardiac evaluation after her Turner syndrome diagnosis. Echocardiogram was suspicious for LVNC, which was confirmed on cardiac magnetic resonance imaging.

## 1. Introduction

Turner syndrome is caused by partial or complete absence of one of the two X chromosomes. It has an incidence of about 40 per 100,000 live births [1]. Congenital heart disease (CHD) occurs in 22–70% of patients with Turner syndrome, and the commonly reported ones include bicuspid aortic valve, coarctation of aorta, partial anomalous pulmonary venous connection, atrial and ventricular septal defects, pulmonary valve abnormalities, patent ductus arteriosus, persistent left superior vena cava, and interrupted inferior vena cava. Severe left-sided cardiac malformations in Turner conceptions are thought to result in early abortions. Studies to look at the myriad of genetic regulatory mechanisms leading to these cardiac defects are ongoing [2].

Left ventricular noncompaction (LVNC) is a morphological abnormality of the left ventricular myocardium characterized by the presence of a meshwork of myocardial strings interlacing in arrangement. Its gross anatomical appearance is characterized by numerous, excessively prominent trabeculations and deep intertrabecular recesses. Echocardiography (ECHO) is used for initial diagnosis and monitoring. The most practiced ECHO criteria for LVNC include segmental thickening of myocardial wall of the left ventricle with two layers: a thin epicardial layer and a thick endocardial layer with prominent trabeculations and deep recesses; absence of other congenital heart diseases; the ratio of noncompacted myocardium to compact myocardium at the end of systole is >2 : 1; the trabeculae are usually located on the apical/lateral, middle/bottom walls of the left ventricle; most noncompacted segments are hypokinetic; and the flow between the intertrabecular recesses can be identified by using the color Doppler method [3]. Difficulty in visualization of apical region on ECHO has made cardiac magnetic resonance imaging (MRI) the preferred method for evaluation of LVNC. Cardiac MRI criteria for the diagnosis include visual appearance of two distinct myocardial layers (a compacted epicardial layer and a noncompacted endocardial layer), marked trabeculation and deep intertrabecular recesses within the noncompacted layer, and noncompacted to compacted end-diastolic myocardial ratio >2.3 and/or noncompacted to global end-diastolic left ventricle mass ratio >0.20 [4]. Risks and clinical outcomes of LVNC are heterogeneous, ranging from no symptoms to arrhythmias and major events such as heart failure, thromboembolism, and sudden cardiac death. LVNC has been previously reported in only three adult patients with Turner syndrome [5–7]. We report the first pediatric case of LVNC diagnosed in a female with Turner syndrome.

## 2. Case Presentation

**Figure 1:**
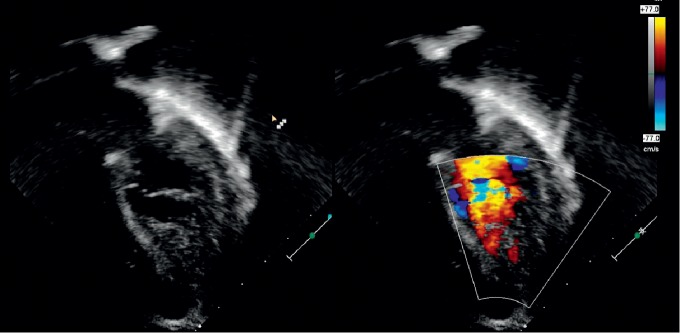
Four-chamber ECHO with color Doppler showing a spongy looking myocardium with dilated sinusoids suggestive of left ventricular noncompaction.

**Figure 2:**
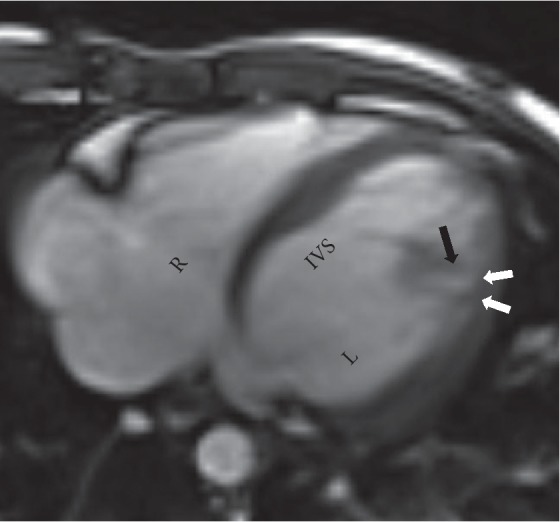
Axial steady-state free precession (SSFP) image depicting thinning of the compacted myocardium (white arrows) and prominent trabeculations along the lateral wall (black arrow) with noncompacted to compacted ratio >2.3.

**Figure 3:**
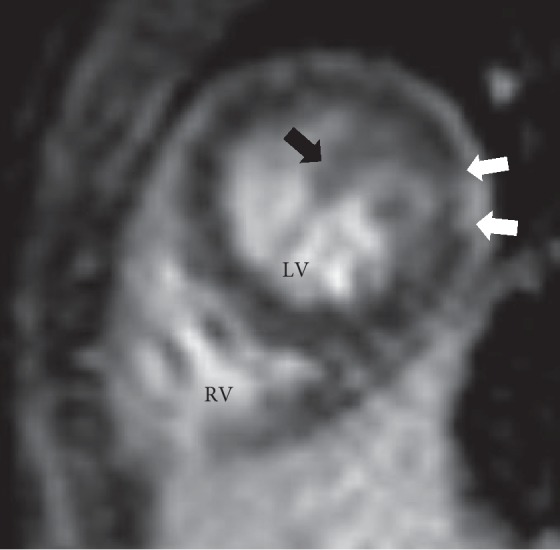
Short axis plane late gadolinium enhancement (LGE) image showing corresponding enhancement in the affected apical myocardium (white arrows) and extensive trabeculations at the ventricular apex (black arrow).

A 6-year-old asymptomatic female who was incidentally diagnosed with mosaic Turner syndrome (XO/XX) during work-up for global developmental delay was referred for cardiac evaluation secondary to her genetic condition. Physical exam was significant for short stature, nevi on skin and a broad based gait. A two-dimensional ECHO was remarkable for severe apical and posterior wall left ventricular hypertrophy with a spongy appearance. An apical four-chamber view with color Doppler was remarkable for red speckles (sinusoids) within the hypertrophied left ventricular muscle (Figure 1) raising the concern for LVNC. The ECHO pictures were not definitive for confirming the diagnosis, so a cardiac MRI was obtained. Cardiac MRI revealed increased trabeculations along the posterior apical aspect of left ventricle with noncompacted to compacted myocardial ratio >2.3 consistent with left ventricular noncompaction (Figures 2 and 3). The patient is being managed conservatively since she is clinically asymptomatic. Her follow-up visits with cardiology show preserved cardiac function (ejection fraction of 54% on the right side and 53% on the left side). She is receiving multidisciplinary care as an outpatient.

## 3. Discussion

LVNC is a genetic cardiomyopathy characterized by left ventricular prominent trabeculations and sinusoids communicating with its cavity. It may be isolated or may occur in conjunction with other CHD such as stenotic diseases of left ventricular outflow tract, Ebstein's anomaly, septal defects, and tetralogy of Fallot [8].

There is no reported genetic association of LVNC with Turner syndrome. To our knowledge, only three cases have been reported in the literature of such an occurrence, who are all adult patients [5–7]. Altenberger et al. described a 45-year-old male with mosaic Turner syndrome who was diagnosed with LVNC when imaging was done during an episode of heart failure and persisted after the heart failure resolved [5]. Erer et al. described a 21-year-old female with Turner syndrome who was diagnosed incidentally when she presented with tachycardia and did not require medical therapy as her ventricular function was intact [6]. Altenberger et al. described another 53-year-old male with mosaic Turner syndrome who was diagnosed with LVNC at 45 years of age, and exhibited regression of LVNC after treatment of heart failure [7]. These cases diagnosed in symptomatic patients in adulthood, in conjunction with incidental diagnosis in our pediatric patient, are suggestive of the need for a higher index of suspicion of LVNC in Turner syndrome patients. Genetic studies reveal LVNC may be linked to X-linked inheritance as evidenced by involvement of tafazzin gene located on chromosome X [9]. This may place patients with Turner's syndrome at a higher risk of developing LVNC. These patients are routinely referred for cardiac evaluation and ECHO to rule out CHD. Further cardiac imaging with MRI or computed tomography angiography may be required if LVNC is suspected but not confirmed by ECHO [10].

Isolated LVNC is strongly associated with arrhythmias and cardiac dysfunction in children. These children are at risk of need for transplant or sudden cardiac death (18.2%) and overall mortality (12.8%) [11]. The risk of these complications may be exemplified in the presence of comorbidities such as Turner syndrome.

## 4. Conclusion

We report the first case of LVNC in an asymptomatic 6-year-old girl with Turner syndrome. Limited cases of this occurrence have been reported in literature. Advanced cardiac imaging is recommended in cases where ECHO is not diagnostic of LVNC. Isolated LVNC has been associated with cardiac complications in the past, and its diagnosis in patients with other comorbidities such as Turner syndrome may require close follow-up.
